# Between illness and hunger: the food crisis affecting patients in Ecuador

**DOI:** 10.3389/fpubh.2025.1702552

**Published:** 2025-11-04

**Authors:** Jorge Vasconez-Gonzalez, Jesus Tamayo, Juan S. Izquierdo-Condoy, Freyce Lema, Esteban Ortiz-Prado

**Affiliations:** One Health Research Group, Universidad de Las Américas, Quito, Ecuador

**Keywords:** food crisis, nutrition, malnutrition, public health, health care system, hospitals

In hospital settings, adequate nutrition contributes not only to patients' physical recovery but also to their psychological wellbeing; therefore, it should be regarded as an essential component of patient care (1). Inadequate nutrition or insufficient food intake during hospitalization leads to multiple adverse outcomes—most notably malnutrition (2). Malnutrition increases the risk of complications such as delayed wound healing, prolonged and costly hospital stays, readmissions, and, in severe cases, death (3). Several studies have confirmed the high prevalence of malnutrition among hospitalized patients. For example, Agarwal et al. analyzed 3,122 patients across 56 hospitals in Australia and New Zealand and found that 32% were malnourished and 23% consumed ≤ 25% of the food provided. Similarly, Doganay et al., in a study of 191,028 patients in Türkiye, reported an overall malnutrition-risk prevalence of 11.6% and 20.4% among patients over 65 years (4, 5). In Ecuador, a study involving 5,355 hospitalized patients across 36 public hospitals in 22 provinces found that 37.1% were affected by malnutrition (6). These data underscore the importance of adequate nutrition for hospitalized patients; however, Ecuador is currently facing food shortages in public hospitals.

During the 1980s and 1990s, health-sector reforms were implemented throughout Latin America, leading to a significant reduction in public health expenditure (7). Although the neoliberal health-sector reform was not formally adopted in Ecuador, several of its core elements were nonetheless present, including drastic cuts to public budgets, the establishment of a universal health insurance scheme with limited coverage for specific population groups, and the outsourcing of services to private providers (7). In 2000, public health expenditure accounted for only 1.5% of GDP. Following the recognition of the right to health in the 2008 Constitution, public spending increased progressively: it reached 1.7% in 2009, exceeded 5% of GDP between 2014 and 2016, and peaked at 5.28% in 2021. Nevertheless, by 2022, public health expenditure had declined again to 4.9% of GDP (8).

In recent years, Ecuador's public healthcare system has faced profound challenges that have culminated in a health crisis. Food shortages have been reported in public hospitals across the country, largely attributable to budget cuts implemented between 2023 and 2025. During this period, the Ministry of Health's budget decreased from USD 3.2 billion to USD 2.7 billion, while allocations for food, security, and cleaning services in public hospitals were reduced from USD 115.8 million to USD 37.2 million—a 67.8% decrease, equivalent to USD 78.6 million (9, 10). In addition to the budget reduction, allocated funds often arrive late or only in small installments (11). As a result, public hospitals have accumulated unpaid debts to food supplier (12), some reportedly amounting to approximately USD 60,000 (11).

Hospitals in Quito, Guayaquil, Ambato, Santo Domingo, and Orellana have reported severe difficulties in providing meals to patients. On several occasions, patients have not received complete meals and have been forced to rely on donations (13–15). Some large hospitals, including those with over 400 beds, have turned to food banks to meet their needs. In Quito, for instance, the local food bank has donated more than 1,700 kilograms of chicken, vegetables, and other supplies since June 2025 (10, 16). The crisis also affects healthcare workers, many of whom depend on hospital-provided meals during on-call shifts. Doctors, nurses, and administrative staff have at times brought food themselves to share with patients. Despite these circumstances, hospitals identified by patients and their families as unable to provide adequate nutrition continue to insist that they serve five meals a day and, in some cases, restrict families from bringing food (10, 13).

The shortage of food further weakens Ecuador's already fragile public healthcare system, which also faces persistent shortages of medicines and medical supplies. This is compounded by the government's multimillion-dollar debts to external providers of essential services such as laboratory testing, imaging, and dialysis, many of whom have suspended operations (17–19).

The ongoing food shortages in Ecuador's hospitals constitute a clear violation of patients' right to health. Beyond undermining human dignity, this crisis exacerbates disease progression, delays recovery, and increases financial strain on an already overburdened healthcare system. Despite the gravity of the situation, the response from the relevant authorities has been markedly insufficient. If health has ceased to improve and health inequalities have worsened, it suggests that society as a whole has stopped progressing and that social inequalities have also deepened (20). Therefore, the ethics of production processes and economic actions must be regarded as a shared sphere of responsibility, encompassing both private actors and the state, including governmental institutions (21).

Immediate and coordinated action is imperative. Ensuring food security in public hospitals must be prioritized through short-term emergency measures and complemented by sustainable long-term policies—such as strengthening food-procurement processes, implementing legislative reforms, and maintaining partnerships and agreements with food-supply companies to ensure a robust, flexible, and sustainable supply chain. Furthermore, urgent intersectoral collaboration—encompassing health, social protection, finance, and civil society—is needed to restore the integrity of patient care and safeguard public health.
